# Eruptive Xanthomas Precipitated by Severe Hypertriglyceridemia From Diabetes and Alcohol Use

**DOI:** 10.7759/cureus.43288

**Published:** 2023-08-10

**Authors:** Nicholas R Munoz, Chibuike C Agwuegbo, Fatima Gauhar

**Affiliations:** 1 Internal Medicine, Temecula Valley Hospital, Temecula, USA; 2 Internal Medicine, Southwest Healthcare, Temecula, USA; 3 Internal Medicine, Riverside Medical Clinic, Temescal Valley, USA

**Keywords:** hypertriglyceridemia (tg), hypertriglyceridemia, skin lesion biopsy, histology and histopathology, adult dermatology, diabetes type ii, alcohol use, triglyceride, hyperlipidemia treatment, eruptive xanthoma

## Abstract

Hypertriglyceridemia is a common abnormality found in patients in the clinical setting. Severe hypertriglyceridemia may manifest phenotypically as eruptive xanthomas, which are red or yellow papules found on the skin, most commonly on extensor surfaces and buttocks. We present a case of severe hypertriglyceridemia in a patient found in the outpatient setting, which manifested as eruptive xanthomas in his posterior upper arms, back, buttocks, axilla, and legs. Laboratory testing of his lipid profile revealed extremely high triglyceride levels, and the patient was immediately referred to the nearest hospital where he was admitted to the intensive care unit (ICU). He was promptly managed with a low-fat diet, anti-hypertriglyceridemic agents, and insulin infusion, with a dramatic reduction in triglyceride levels. He subsequently underwent a skin biopsy which confirmed the diagnosis of eruptive xanthoma. Rapid reduction in triglyceride levels is instrumental in the prevention of complications, most notably, acute pancreatitis. This article highlights the importance of a high index of suspicion for recognition of the signs and symptoms of severe hypertriglyceridemia, as well as the different management options available for the control of triglyceride levels and the prevention of complications.

## Introduction

Hypertriglyceridemia is one of the most common laboratory abnormalities in clinical practice. The cause of high triglyceride levels is often multifactorial, attributed to genetic factors, diet, exercise level, smoking history, obesity, and drugs [1]. Severe hypertriglyceridemia can be associated with skin lesions called eruptive xanthomas. Eruptive xanthomas are red or yellow papules ranging in size from 1 to 3 mm. They usually form in groups most commonly on extensor surfaces and buttocks. They are almost always associated with hypertriglyceridemia; often triglyceride levels exceed 1,500 mg/dL in the presence of eruptive xanthomas [2]. Triglycerides greater than 1,000 mg/dL are considered severe hypertriglyceridemia and are a high risk for pancreatitis [3]. The case of a 39-year-old with a history of type 2 diabetes mellitus and mixed hyperlipidemia who presented with numerous erythematous skin papules is presented. The papules were subsequently diagnosed as eruptive xanthoma after a serum triglyceride level of 6,284 mg/dL resulted. The patient was treated in the ICU with an insulin drip.

## Case presentation

The patient is a 39-year-old male with a past medical history of mixed hyperlipidemia and type 2 diabetes mellitus who presented with multiple skin lesions. On a previous visit, his A1C was found to be 10.1%, cholesterol 200 mg/dL, and triglycerides 395 mg/dL. The patient was an alcohol user who endorsed drinking five to seven beers per day on weekdays and 13 beers a day on weekends. After his diagnosis of diabetes, the patient reported he ate a healthier diet consisting of vegetables, chicken, and salmon. He reported a 22-year smoking history, smoking one to two cigarettes per day. He was overweight with a BMI of 29.8. His home medications included metformin 500 mg twice daily, losartan 25 mg daily, rosuvastatin 5 mg daily, and omeprazole 40 mg daily.

**Figure 1:**
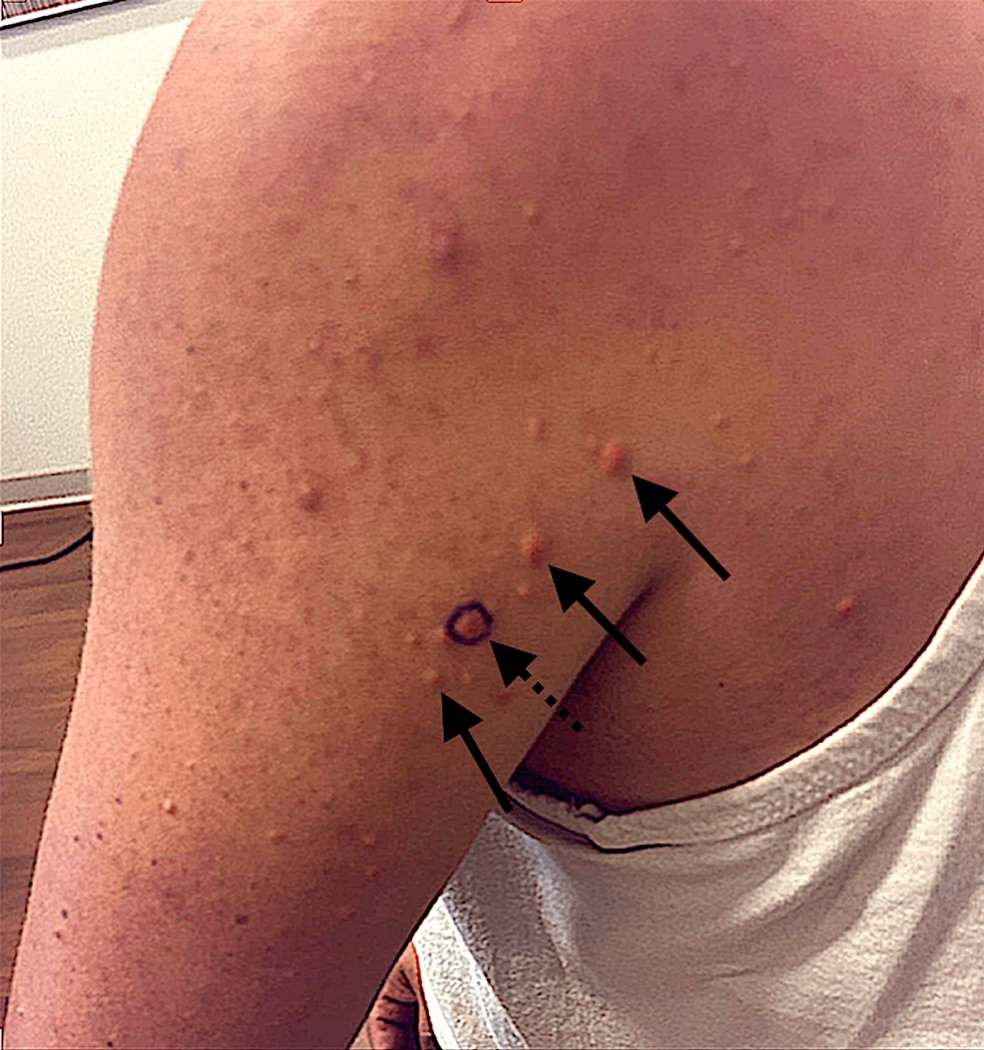
Eruptive xanthomas on the patient's left posterior proximal arm. The papules in the image were found to be eruptive xanthomas (arrows). The dotted arrow represents a xanthomatous nodule that was circled with a marker to track its growth.

**Figure 2:**
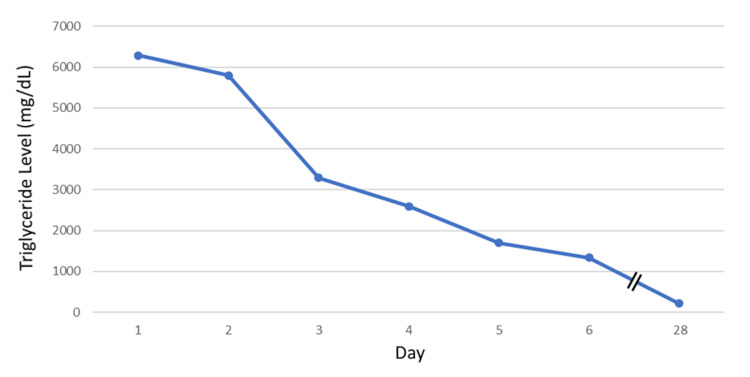
The patient's triglyceride levels the day before (day 1), during (days 2-6), and after (day 28) hospitalization.

On physical exam, the patient was found to have numerous 1-2 mm erythematous papules on the posterior upper arms, back, buttocks, axilla, and below the knees bilaterally (Figure 1). The patient stated the lesions appeared 10 days previously and were associated with mild pain on palpation. He denied other symptoms such as fever, chills, pruritus, bleeding, and recent illness. Based on their appearance, the differential diagnosis for the patient's lesions included nodular acne and eruptive xanthomas. He was given a prescription for doxycycline as the lesions appeared to be nodular acne, an urgent referral to dermatology, and labs including a lipid panel to rule out eruptive xanthomas. The following day, the lipid panel resulted in an extremely high triglyceride level of 6,284 mg/dL, cholesterol levels of 795 mg/dL, and high-density lipoprotein (HDL) of 11 mg/dL. He was found to have a glucose level of 380 g/dL and an A1C of 9.7%. The patient was immediately contacted with the laboratory results and subsequently complained of new-onset epigastric abdominal pain. Due to the patient's high risk for pancreatitis, he was instructed to present to the nearest hospital emergency department. The patient presented to his local hospital and was admitted to the ICU. In the ICU, his triglyceride level was initially 5,873 mg/dL, blood glucose was 420 g/dL, his AST was 93 U/L, ALT 91 U/L, and a lipase level was normal at 123 U/L. The patient was started on an insulin drip, fenofibrate 160 mg daily, a low-fat carbohydrate-controlled diet, and omega-3 acid ethyl esters 1 g daily. He had a bowel movement in the hospital, and his abdominal pain resolved. After three days, the patient's triglycerides decreased to 1,337 mg/dL, the insulin drip was discontinued, and he started 10 units of insulin glargine daily (Figure 2). He was discharged the following day with instructions to continue his medications including 10 units of insulin glargine daily, fenofibrate 160 mg daily, rosuvastatin 5 mg daily, omega-3 acid ethyl esters 1 g daily, and metformin 500 mg twice daily. A lower dose of rosuvastatin was prescribed as the patient had elevated liver function tests.

**Figure 3:**
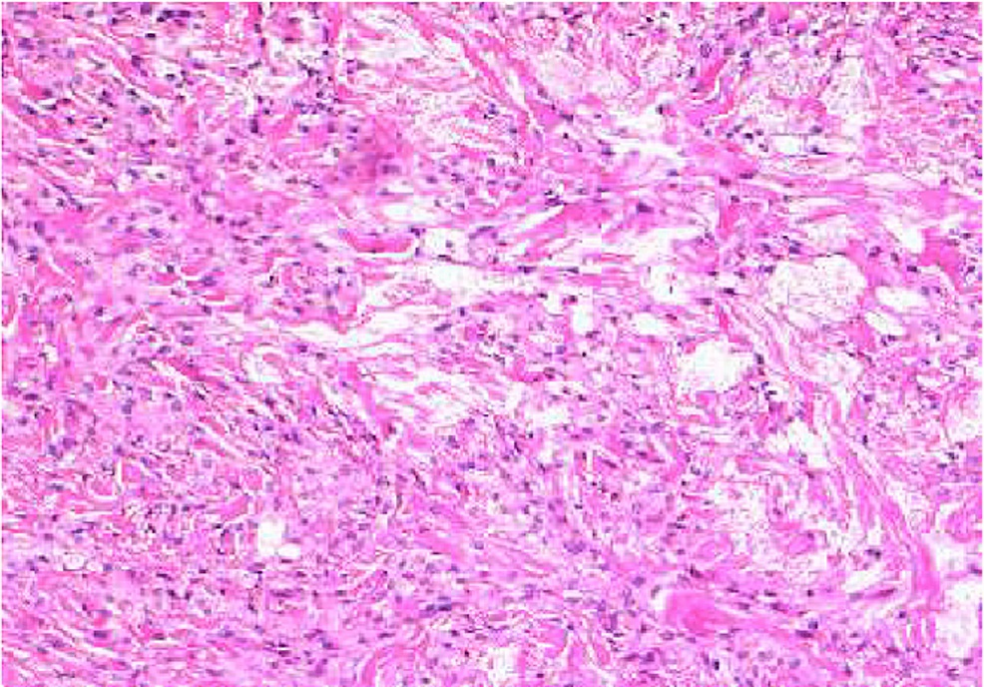
Hematoxylin and eosin stain of a punch biopsy of a xanthoma on the patient's left posterior arm. The stain shows discrete collections of foamy histiocytes throughout the dermis associated with a slight fibroplasia. This specimen was diagnosed as a xanthoma by the pathologist.

After hospitalization, the patient was seen by dermatology who performed a biopsy of the lesions. The biopsy showed discrete collections of foamy histiocytes present throughout the dermis in association with a slight fibroplasia consistent with xanthoma (Figure 3). Three weeks after discharge, the patient's blood glucose was found to be elevated at 161 mg/dL with a hemoglobin A1C of 10.0. His repeat triglyceride level was 213 mg/dL, and his cholesterol was 154 mg/dL (Table 1). His insulin glargine was increased to 16 units daily, his metformin was increased to 1,000 mg twice daily, rosuvastatin was increased to 20 mg daily, and fenofibrate 160 mg daily was continued. He reported a decrease in alcohol intake to 4 oz of vodka once a week after hospital discharge.

**Table 1:** The patient's lipid levels, serum glucose, and hemoglobin A1C levels in the days and months surrounding hospitalization. *Triglyceride level was too high for an accurate VLDL or LDL estimation. HDL: high-density lipoprotein, VLDL: very-low-density lipoprotein, LDL: low-density lipoprotein.

	20 days after hospital discharge	1 day before hospitalization	6 months before hospitalization
Triglycerides	213 mg/dL	6,283 mg/dL	395 mg/dL
Cholesterol	154 mg/dL	795 mg/dL	200 mg/dL
HDL	38 mg/dL	11 mg/dL	37 mg/dL
VLDL	36 mg/dL	*	66 mg/dL
LDL	80 mg/dL	*	97 mg/dL
Glucose	161 mg/dL	380 mg/dL	210 mg/dL
A1C	10.0%	9.7%	10.1%

## Discussion

The patient presented likely had eruptive xanthomas due to a combination of lifestyle and genetic factors. He was an active smoker, had uncontrolled diabetes, a history of elevated triglycerides, and excessive alcohol use. The patient's uncontrolled diabetes combined with his heavy alcohol use was the major factor causing the hypertriglyceridemia and eruptive xanthomas. Alcohol use is a major risk factor for hypertriglyceridemia as alcohol is a substrate for triglyceride production. Alcohol increases the production of fatty acids in the liver increasing very-low-density lipoprotein (VLDL) triglyceride secretion [3].

The patient likely had a genetic component of his hypertriglyceridemia. Confirmation of a genetic etiology of hypertriglyceridemia does not impact management; therefore, genetic testing remains controversial as the benefit is unclear [4]. As it would not affect the management of his hypertriglyceridemia, genetic testing was not performed for the patient.

Xanthomas are lipids that accumulate in foam cells of the dermis [5]. There are different types of xanthomas such as tendinous xanthomas which are skin-colored nodules that are usually firm and mobile. They occur most commonly over the Achilles tendon. Tendinous xanthomas can be observed to move during limb extension and flexion as they are directly attached to the tendon [6]. Plane xanthomas are characterized by yellow-orange plaques usually in the neck, upper thorax, and around the eyes. They have been associated with diseases such as multiple myeloma [7]. Verruciform xanthomas are considered a benign neoplasm commonly affecting the oral mucosa [8]. Finally, eruptive xanthomas are small yellow or yellow-red papules usually on the extensor surfaces of limbs or buttocks often caused by hypertriglyceridemia [1].

Eruptive xanthomas are rare, with an estimated prevalence of 18 in 100,000 persons [9]. One study found that among patients presenting with a triglyceride level greater than 20 mM (1,770 mg/dL), 8.5% had eruptive xanthomas [10]. The physiology of eruptive xanthomas caused by hyperlipidemia is not well understood. About 70% of triglyceride in the blood is carried by chylomicrons, a lipoprotein. During severe hypertriglyceridemia, its postulated lipoprotein extrusion through capillary blood vessels in the dermis leads to the uptake of lipoproteins in peripheral macrophages. This uptake leads to the foam cells seen on histology in eruptive xanthomas [5].

The initial approach to the management of mild to moderate hypertriglyceridemia is lifestyle modification, including diet changes and weight loss. A diet low in carbohydrates, added sugars, refined starch, and saturated fats is encouraged. These should be substituted with a diet high in fruits, unsaturated fat, whole grains, and legumes. Alcohol has been well recognized as a contributory factor to hypertriglyceridemia and therefore should be avoided in at-risk patients [11]. Weight loss of 10% reduces the triglyceride level by approximately 20% [12]. Certain medications cause an increase in triglyceride levels, including thiazides, estrogen, second-generation antipsychotic agents, corticosteroids, and propofol. These should be avoided if possible. When lifestyle modification fails to reduce triglyceride levels, pharmacological intervention is required [13]. Medications that preferentially target serum triglycerides include omega-3 fatty acids, fibrates, and niacin. Omega-3 fatty acids include alpha-linolenic acid, eicosapentaenoic acid, and docosahexaenoic acid, which can be found naturally in foods such as seafood, plant oils, flaxseed, and soybean. Prescription omega-3 fatty acids in combination with statins have been found to be beneficial in reducing all-cause mortality in patients with established cardiovascular disease [14]. Fibrates are peroxisome proliferator-activated α-receptor (PPAR-α) agonists which have been shown to reduce triglyceride levels most effectively among all the pharmacological agents available. Niacin is more potent in raising high-density lipoprotein cholesterol (HDL-C) levels; therefore, they are the drug of choice in patients with high triglycerides and low HDL-C [15]. Statins, ezetimibe, and proprotein convertase subtilisin/kexin 9 (PCSK9) monoclonal antibodies are less potent in lowering triglycerides. However, they are recommended for lowering low-density lipoprotein cholesterol (LDL-C) levels as well as reducing the atherosclerotic cardiovascular disease (ASCVD) risk [16].

In patients with severe hypertriglyceridemia, prevention of complications, specifically acute pancreatitis, is the primary goal. Patients should be started on a low-fat diet, and concurrent pharmacological interventions using omega-3 fatty acids, fibrates, or niacin are necessary in preventing acute pancreatitis and reducing triglyceride levels to below 500 mg/dL. Once this has been achieved, ASCVD risk reduction using LDL-C-lowering medications becomes the main goal [12].

Acute pancreatitis is the main complication of severe hypertriglyceridemia. In such patients, treatment begins with diet restriction including bowel rest, nil per oral, or a very low-fat diet. Intravenous fluid therapy and pain control are essential in the acute setting. Insulin infusion and plasma exchange have been used in the treatment of acute pancreatitis; however, their use is still controversial and should be decided on an individualized basis. These interventions are employed in an attempt to rapidly lower serum triglyceride levels and therefore reduce the burden of inflammation in pancreatitis. Insulin activates lipoprotein lipase thereby leading to the degradation of chylomicrons, which leads to the reduction of serum triglycerides. There have been case reports describing the successful use of insulin in acute pancreatitis, but there have been no studies or trials comparing insulin to conservative therapy [17]. Heparin has been used in combination with insulin in acute pancreatitis to acutely lower triglyceride levels [18]. There are also no trials or studies that directly compare heparin with other treatment modalities. The concern with heparin is rebound hypertriglyceridemia due to the eventual depletion of vascular endothelial lipoprotein lipase. Plasmapheresis rapidly removes triglycerides, chylomicrons, and inflammatory mediators from the serum, thereby reducing inflammation [17]. While plasmapheresis has been successfully used in case reports, prospective and retrospective studies have failed to show any mortality benefit when compared to conservative management [19]. One multicenter retrospective study did, however, demonstrate the usefulness of plasmapheresis in patients with hypertriglyceridemia who were resistant to lifestyle modifications and pharmacological therapy [20].

## Conclusions

This article highlights a patient with nodular skin lesions that were found to be eruptive xanthomas. Alcohol use and uncontrolled diabetes were the major contributors to the patient's hypertriglyceridemia. Eruptive xanthomas should be considered in patients presenting with risk factors and characteristic skin lesions. In a patient with skin lesions that may be eruptive xanthoma, a lipid panel should be ordered. Clinicians should consider hypertriglyceridemia in patients with risk factors, such as heavy alcohol use, who present with skin lesions that are similar in appearance to eruptive xanthomas.

## References

[1] Simha V (2020). Management of hypertriglyceridemia. BMJ.

[2] Nayak KR, Daly RG (2004). Eruptive xanthomas associated with hypertriglyceridemia and new-onset diabetes mellitus. N Engl J Med.

[3] Berglund L, Brunzell JD, Goldberg AC, Goldberg IJ, Sacks F, Murad MH, Stalenhoef AF (2012). Evaluation and treatment of hypertriglyceridemia: an Endocrine Society clinical practice guideline. J Clin Endocrinol Metab.

[4] Deshotels MR, Hadley TD, Roth M (2022). Genetic testing for hypertriglyceridemia in academic lipid clinics: implications for precision medicine: brief report. Arterioscler Thromb Vasc Biol.

[5] Parker F, Bagdade JD, Odland GF, Bierman EL (1970). Evidence for the chylomicron origin of lipids accumulating in diabetic eruptive xanthomas: a correlative lipid biochemical, histochemical, and electron microscopic study. J Clin Invest.

[6] Tsouli SG, Kiortsis DN, Argyropoulou MI, Mikhailidis DP, Elisaf MS (2005). Pathogenesis, detection and treatment of Achilles tendon xanthomas. Eur J Clin Invest.

[7] Cohen YK, Elpern DJ (2015). Diffuse normolipemic plane xanthoma associated with monoclonal gammopathy. Dermatol Pract Concept.

[8] Shafer WG (1971). Verruciform xanthoma. Oral Surg Oral Med Oral Pathol.

[9] Leaf DA (2008). Chylomicronemia and the chylomicronemia syndrome: a practical approach to management. Am J Med.

[10] Sandhu S, Al-Sarraf A, Taraboanta C, Frohlich J, Francis GA (2011). Incidence of pancreatitis, secondary causes, and treatment of patients referred to a specialty lipid clinic with severe hypertriglyceridemia: a retrospective cohort study. Lipids Health Dis.

[11] Stone NJ (1994). Secondary causes of hyperlipidemia. Med Clin North Am.

[12] Hernandez P, Passi N, Modarressi T (2021). Clinical management of hypertriglyceridemia in the prevention of cardiovascular disease and pancreatitis. Curr Atheroscler Rep.

[13] Mach F, Baigent C, Catapano AL (2020). 2019 ESC/EAS Guidelines for the management of dyslipidaemias: lipid modification to reduce cardiovascular risk. Eur Heart J.

[14] Bhatt DL, Steg PG, Miller M (2019). Cardiovascular risk reduction with icosapent ethyl for hypertriglyceridemia. N Engl J Med.

[15] Oh RC, Lanier JB (2007). Management of hypertriglyceridemia. Am Fam Physician.

[16] Santos-Baez LS, Ginsberg HN (2020). Hypertriglyceridemia: causes, significance, and approaches to therapy. Front Endocrinol (Lausanne).

[17] Garg R, Rustagi T (2018). Management of hypertriglyceridemia induced acute pancreatitis. Biomed Res Int.

[18] Jain D, Zimmerschied J (2009). Heparin and insulin for hypertriglyceridemia-induced pancreatitis: case report. ScientificWorldJournal.

[19] Gubensek J, Buturovic-Ponikvar J, Romozi K, Ponikvar R (2014). Factors affecting outcome in acute hypertriglyceridemic pancreatitis treated with plasma exchange: an observational cohort study. PLoS One.

[20] Stefanutti C, Di Giacomo S, Vivenzio A (2009). Therapeutic plasma exchange in patients with severe hypertriglyceridemia: a multicenter study. Artif Organs.

